# Development and evaluation of a computer-based medical work assessment programme

**DOI:** 10.1186/1745-6673-3-35

**Published:** 2008-12-18

**Authors:** Stefanie Mache, Cristian Scutaru, Karin Vitzthum, Alexander Gerber, David Quarcoo, Tobias Welte, Torsten T Bauer, Michael Spallek, Andreas Seidler, Albert Nienhaus, Burghard F Klapp, David A Groneberg

**Affiliations:** 1 Institute of Occupational Medicine, Charité – Universitätsmedizin Berlin, Free University and Humboldt University, Thielallee 69-73, 14195 Berlin, Germany; 2 Department of Respiratory Medicine, Hannover Medical School, Carl-Neuberg-Straße 1, 30625 Hannover, Germany; 3 Department of Medicine/Psychosomatics, Charité – Universitätsmedizin Berlin, Free University and Humboldt University, Luisenstrasse 13a, 10117 Berlin, Germany; 4 The Federal Institute for Occupational Safety and Health, Nöldnerstr. 40-42, 10317 Berlin, Germany; 5 City Hospital Emil von Behring/Lungenklinik Heckeshorn, Walterhöferstrasse 11, 14165 Berlin, Germany; 6 Institution for Statutory Accident Insurance in the Health and Welfare Services, Pappelallee 35/37, 22089 Hamburg, Germany

## Abstract

**Background:**

There are several ways to conduct a job task analysis in medical work environments including pencil-paper observations, interviews and questionnaires. However these methods implicate bias problems such as high inter-individual deviations and risks of misjudgement. Computer-based observation helps to reduce these problems. The aim of this paper is to give an overview of the development process of a computer-based job task analysis instrument for real-time observations to quantify the job tasks performed by physicians working in different medical settings. In addition reliability and validity data of this instrument will be demonstrated.

**Methods:**

This instrument was developed in consequential steps. First, lists comprising tasks performed by physicians in different care settings were classified. Afterwards content validity of task lists was proved. After establishing the final task categories, computer software was programmed and implemented in a mobile personal computer. At least inter-observer reliability was evaluated. Two trained observers recorded simultaneously tasks of the same physician.

**Results:**

Content validity of the task lists was confirmed by observations and experienced specialists of each medical area. Development process of the job task analysis instrument was completed successfully. Simultaneous records showed adequate interrater reliability.

**Conclusion:**

Initial results of this analysis supported the validity and reliability of this developed method for assessing physicians' working routines as well as organizational context factors. Based on results using this method, possible improvements for health professionals' work organisation can be identified.

## Background

In general, job task analysis can be defined as the study to identify and typify the fundamental characteristics of a specific work-related activity or set of activities. It is a methodology supported by a number of techniques to help an analyst capture meaningful, quantitative data about how and where employees spend their time [1,2].

These techniques can include simple observations, interviews as well as video documentation. In former studies, questionnaires were preferentially used or the traditional method of stopwatch, pencil and protocol sheet [3].

But there are some limitations in using these methods: Pencil-and-paper data collection is time consuming and imprecise [4]. Likewise, reduced reliability is a problem of these conventional methods [5]. Remembering working events or examples of behaviour during subjective interviews can present problems of bias [6].

Equally, the exclusive use of questioning causes other risks such as the risk of misjudgement and inadequate confrontation with real working events [1].

Computer-based recording methodology reduces these problems. Different tasks can be collected precisely to the nearest second. In this way, inter-observer reliability can be optimised.

This paper illustrates data pertaining to this development, reliability and validity of a job task analysis instrument for monitoring physicians' job tasks in different medical settings. This effort is part of a larger study to analyse working behaviours aiming to identify potential improvements for health professionals' work efficiency.

## Methods

### Classification of job tasks

The first step in developing the taxonomy was to generate lists of tasks what physicians perform in different care settings (Internal Medicine, Paediatrics, Neurology, Surgery and Psychiatrics). To obtain these, a literature review was done for job tasks performed in these medical areas. Afterwards interviews were conducted with experienced specialists in these areas.

### Content validity of the job task classification

Content validity of the task lists were confirmed by all experienced specialists in each medical area. The final classification incorporated following conditions: 1. the tasks should not have any common characteristics (exclusiveness) and 2. all performed clinical tasks should be captured exhaustively. Additionally, the classification should be easy to handle in training and using.

Observations, ranging from 3 to 5 work shifts per hospital department (Internal Medicine, Paediatrics, Neurology, Surgery and Psychiatrics) took place to prove content validity of the tasks.

After this observation process, researchers and physicians modified the lists. Adjustments were made in terms of task name changes, reorganization of categories and additions or deletions of tasks. After all task lists were verified for completeness and accuracy they were implemented in the data collection software (see below).

### Development of the job task analysis instrument

Database-linked object oriented software was developed with Borland's CBuilder. The different job tasks are coded and saved in a database, thus allowing changes for supporting different medical settings. They are sorted into categories, which are presented by the software as different tabs. Each tab contains the corresponding job tasks. The linkage between tasks and tabs is held up in the database preserving the flexibility of the system.

The acquired data is saved in different text files. When a task change occurs, the implemented logic in the software operates the changes and saves the event. Sophisticated software filters protect the software from false inputs (e.g. simultaneous main tasks).

**Figure 1:**
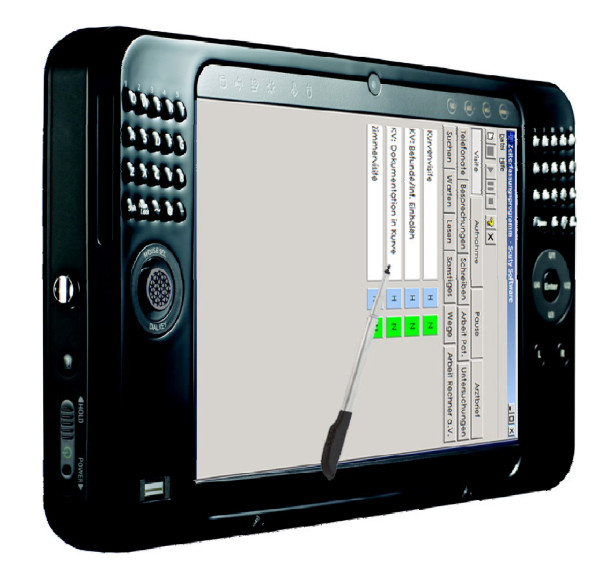
Ultra mobile PC.

The developed software was implemented in an Ultra mobile PC (UMPC). An UMPC is a small handheld laptop with a pressure-sensitive screen (see figure 1). It measures 3 × 12 × 23 cm and can be carried in one hand. Use of a special stylus, included with the UMPC, allows the observer to operate the computer applications. As it should be easy for observers to identify various job tasks, the screen was designed user friendlier by varying the column widths, colours and fonts. Symbolisation helps to develop easy running processes and guarantees legibility and comparability of the noted expiries.

### Evaluation of inter-observer reliability

Identifying and recording job tasks often rely to some degree upon the subjective interpretations of observers. Therefore, this study should include statistics measuring the agreement between two observers. To address this, statistical measurements of inter-observer agreement was required.

As the task classification and data collection instrument were complete, the researchers began to conduct observations to evaluate inter-observer reliability.

Two trained researchers recorded simultaneously tasks of the same physician during six-hour observation periods in five medical hospital departments (Internal Medicine, Paediatrics, Neurology, Surgery and Psychiatrics). Each researcher stood in such a position to be able to observe the physician's activities precisely, while remaining unable to view the screen of the other researcher's UMPC. Since discussion of data or the data collection process could bias reliability measures, researchers were obliged not to communicate with each other during the observation.

**Figure 2:**
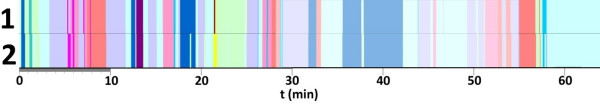
**Evaluation of inter-observer reliability**. 1 – first observer; 2 – second observer

The text data file (in Microsoft Excel format) of each researcher's observation was imported into the programme file. The programme analysed each line of the data, searching for corresponding tasks between researchers (see figure 2). Criteria for congruency between tasks were implemented using the two variables of specific task type and time. Each time the programme identified an agreement between the researchers' observations, the line was selected as a 'hit'. A 'miss' resulted if both researchers recorded the same task but with a time delay of more than 5 seconds or if they observed totally different tasks. Results show the percentage of hits out of the total number of hits and misses during the observation period.

## Results

A new job task analysis instrument was developed allowing trained observers to record medical work activities in real-time via direct observation.

### Application of the job task analysis tool

**Figure 3:**
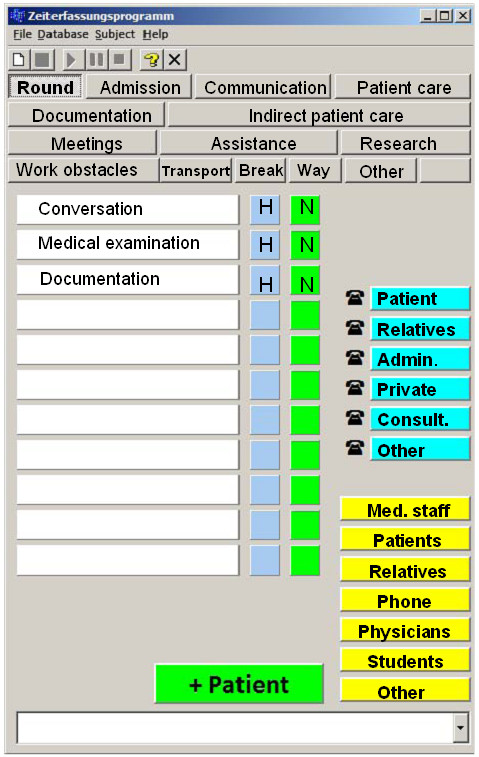
Categorisation system.

The time recording software translates the job task categories from the Visual Basic data bank and generates a category tab for every element of this group. Single activities are added in a list accordingly to the matching activity group. For example, under the category 'administration', activities might include 'documentation', consulting files, general bureaucratic activities etc. (see figure 3).

When beginning an observation, an initial data entry screen prompts the observer to enter participant demographic information (e.g., sex and shift). The date and time when the observations started are automatically recorded and saved by the programme. At this time, the recording of tasks can be started by pressing a tab, for example "Rounds". At that moment the stopwatch starts to run incessantly until the observer finishes the capture (see table 1).

**Table 1:** Extract of time registration

Index card	Action	Starting time	Ending
11	108	08:04:00	08:06:56

12	113	08:06:56	08:07:06

11	108	08:07:06	08:07:09

12	113	08:07:09	08:07:32

8	100	08:07:32	08:07:34

8	102	08:07:34	08:07:34

8	100	08:07:34	08:14:58

5	94	08:14:58	08:15:13

6	95	08:15:13	08:15:44

5	94	08:15:44	08:15:49

6	95	08:15:49	08:15:55

5	94	08:15:55	08:16:19

8	100	08:16:19	08:18:52

12	113	08:18:52	08:18:55

8	100	08:18:55	08:19:13

5	94	08:19:13	08:20:24

6	95	08:20:24	08:20:45

5	94	08:20:45	08:21:10

6	95	08:21:10	08:21:24

5	94	08:21:24	08:21:53

6	95	08:21:53	08:22:00

5	94	08:22:00	08:22:14

6	95	08:22:14	08:22:20

5	94	08:22:20	08:22:26

6	95	08:22:26	08:22:34

5	94	08:22:34	08:23:07

12	113	08:23:07	08:23:21

7	111	08:23:21	08:23:44

8	100	08:23:44	08:24:17

7	111	08:24:17	08:24:27

12	113	08:24:27	08:24:33

5	94	08:24:33	08:25:00

The observer selects the suitable category out of the mentioned list of tabs. At this moment the software stores the combination tab – activity – time (stamp) in a file. The assessed categories are now automatically saved combined with a time code. Each registered task is automatically time-stamped and implemented in a data file. The time stamp is accurate to the second and synchronised continuously with the computer clock. Therefore, a very exact capture is guaranteed. Each time the physician starts a new work activity, the researcher chooses that activity from the task list on the screen.

Tasks implemented in the software can be registered as sequential or as simultaneous. In case of multitasking, the two simultaneous activities are recorded in the moment of their appearance (see table 2). If one running activity is not finished yet, the second activity can easily be recorded according to the protocols. If the primary activity is finished, the former second activity becomes the primary. Further activities are registered as an incidental activity.

**Table 2:** Main and second activities

**Status**	CA1	CA	MA1, SA	MA, SA1	MA, SA	MA, SA	MA, SA
**Event**	+CA2	+SA	+MA2	+ SA2	+CA	-SA	-MA

**Result**	CA1 stop	CA → MA	MA1 stop	SA1 stop	MA stop	SA stop	MA stop
	CA2 start	SA start	MA2 start	SA2 start	SA stop	MA → CA	SA stop
					CA start		SA → CA CA start

CA = Central Activity (no second activity).

MA = Main Activity.

SA = Second Activity.

At the end of the capture the button "Stop" has to be hit. At this moment all events of the observation are automatically saved in a tab-delimited file.

When the assessment is complete, data from each case is transferred to a PC and evaluated statistically and graphically: the number of individual occurrences of each task, mean duration of each occurrence of each task, the total time (in seconds) spent on each task category over the entire observation period, the percentage of total time spent on each task category and aggregated task groups (e.g. all observation tasks) are counted.

The program allows the user to retrieve files, graph data as well as write and print reports or graphs.

### Reliability study

Five trained researcher pairs recorded tasks during observation sessions each lasting six hours in five medical hospital settings (Paediatrics, Internal Medicine, Psychiatrics, Neurology and Surgery). The mean inter-observer reliability was 80% (range = 71% – 86%).

## Discussion

Results from the analysis showed, this job task methodology is having a sufficient validity and reliability in observing physicians in different medical settings [7].

Especially the reliability outcomes proved a high level of similarity – an important outcome measure for the job task analysis. The process of developing the software and the task lists would likely be supportive for other studies since this job task analysis instrument could be extended to other contexts (e.g. offices or factories) as well as other professions (e.g. nurses).

Use of this job task analysis programme allows the acquisition of information which cannot be collected by any other method. It provides a clear picture of physicians' routine. This task analysis method can be used to identify and develop explanations of individual differences in task performance as well as among hospitals and other medical professions.

As a point of criticism, using this methodology is very time and effort intensive; observational data contains an extremely wide amount of information. The more information the researcher desires to collect, the longer the observations and data collection will last. Secondly, direct observation can be an intrusive and disturbing technique. The presence of an observer may directly influence the physician's behaviour [8]. To minimize the possibility of affecting behaviour, the observer should stand at a defined distance from the physician.

## Conclusion

In conclusion, precise data assessment is a complex task, especially in the field of medical work routine. Computer technology can support the collection of such data. A computer-based job task activity programme was developed and evaluated to analyse physicians' working behaviours. Based on results, medical work routines as well as organizational context factors can be examined with a perspective to identify suggestions for improvements for health professionals' work organization.

## Competing interests

The authors declare that they have no competing interests.

## Authors' contributions

SM and DAG designed the study. SM and CS constructed the computer programme. SM wrote the manuscript. SM, CS, TW, AG, KV, DQ, TTB, MS, AS, AN, BFK and DAG participated in the development process, discussion and manuscript writing.

## References

[B1] Ulich E, Ulich E (2005). Arbeitspsychologie. Arbeitspsychologie.

[B2] Salvendy G (1997). Handbook of Human Factors and Ergonomics.

[B3] Fine SA, Cronshaw SF (1999). Functional job analysis: A foundation for human resources management.

[B4] De Leeuw E, Nicholls W (1996). Technological Innovations in Data Collection: Acceptance, Data Quality and Costs. Sociological Research Online.

[B5] Bellack AS, Hersen M (1998). Behavioral assessment.

[B6] Emerson E, Reeves DJ, Felce D, T Thompson DF, Symons FJ (2000). Palmtop computer technologies for behavioral observation research. Behavioral observation: Technology and applications in developmental disabilities.

[B7] Altman D (1991). Practical statistics for medical research.

[B8] Heppner PP, Wampold BE, Kivlighan DM (2008). Research Design in Counseling Thomson.

